# Empowering health: exploring the vital role of facilitator organisations in supporting chronic disease patients in Assam, India

**DOI:** 10.1007/s43999-024-00052-y

**Published:** 2024-10-30

**Authors:** Hiranmoyee Bhuyan, Seema S. Singha

**Affiliations:** Department of Commerce, Dibrugarh University, Dibrugarh, Assam India

**Keywords:** Health care services, Facilitator organization, Non-governmental organization, Chronic disease, Cancer, Health care customer

## Abstract

**Background:**

The research centers on an underrated type of mediator organization namely facilitator organizations (FO) that work in the health care setting. These organizations are mediators that bridge the gap between patients (healthcare customers) and medical services. The facilitator organizations considered for the study are non-governmental organizations situated in Assam that works in align to chronic diseases, particularly cancer.

**Methods:**

The data collection was done through organizational referrals for the study making it a snowball sampling, progressively incorporating additional contemporary entities. The study's respondents were facilitator organizations (i.e.Non-Government Organizations) actively involved in addressing chronic disease. Data were gathered from these facilitator organizations situated in Assam, India, supporting healthcare customers specifically those with cancer. Key informant interviews and semi-structured questionnaires were used for data collection, the responses were documented using a field diary and the Lovelock service model was used as a reference for the construction of the questionnaire and developing the research framework.

**Results:**

The analysis of data shows that facilitator organisations maintain continuity in relationships, which enhances health management and outcomes for customers. The shift in cancer care towards a patient-centred approach and the crucial role of FOs in providing comprehensive and individualized care, addressing diverse patient needs thereby addressing the holistic development of the health care customer is vital. In addition, effective patient-centred communication, incorporating trust, compassion, respect and comprehensive support including mental health therapy, occupational therapy, and rehabilitation plays a crucial role in leading a normal life. `

**Conclusion:**

The facilitator organizations dealing with Cancer have to meet a broad range of services outside the core medical service providers for their healthcare customers. These efforts contribute to the overall recovery of both the healthcare customer with cancer and their family.

## Background

Chronic diseases are conditions that persist for over a year, necessitate continuous medical care, and/or restrict daily activities [60, 65]. According to Harvard Medical School, referencing NHANES data from 1999–2004, the following are categorized as chronic diseases: cardiovascular disease, hypertension, diabetes mellitus, hypercholesterolemia, asthma, COPD, and a history of cancer [9, 63]. One of the most severe consequences for those suffering from chronic illnesses, particularly cancer, is the loss of the ability to perform daily tasks, socialize freely as they once did, and maintain their independence [30]. The detrimental impact of cancer is not only premature death,but also leads to the loss of the economic well-being of individuals, households, and society as a whole. Global Cancer Observatory (GLOBOCAN) predictions for 2020 indicated that 19.3 million cancer cases occurred globally [57]. After China and the United States of America, India came in third place [19, 20]. Globally cancer is one of the primary reason of death as there were 9.7 million cancer-related deaths and about 20 million new cases in 2022 [58]. As per GLOBOCAN, the number of cancer cases in India is expected to rise to 2.08 million by 2040, representing a 57.5 percent increase from 2020. India being a lower middle-income nation, have a disproportionately high cancer incidence rate because of low awareness, limited access to affordable care, and poor prognoses [53]. Also, geographic variations in genetic determinants, environmental exposures, and cancer patterns exist between regions [6]. These differences are driven by diversity in ancestries, socio-economic and cultural attributes, eating habits, and lifestyles [51, 65, 67, 68].

The north eastern states of India, has highest incidence of cancer in the nation and three times more the national average. Although the state of Assam succeeded in controlling leprosy, AIDS, and malaria epidemics, unfortunately still has many miles to go in preventing cancer [18]. According to a 2021 report by the Indian Council of Medical Research [29], the probability of developing cancer over a lifetime is highest in Kamrup Metro, Assam [29]. Additionally, the majority of people in Assam lack health insurance. Contributing factors to this underdeveloped health sector include low literacy rates, low per capita income, a significant disparity between urban and rural areas, and inadequate water and sanitation infrastructure [51]. Although Medical science has made remarkable strides in recent decades, significantly boosting life expectancy in terms of chronic diseases however, this progress has not reached everyone equally in India. With access to basic healthcare hindered by low income, geographic isolation, lack of knowledge etc., families often face difficult decisions [49]. In such scenarios, a facilitator plays a crucial role in navigating these challenges. Weiss et al. [61] discuss "facilitator" and "mediator" as terms encompassing various problem-solving professions. The importance of facilitators spans multiple fields and roles, such as Tutor Facilitators/Educational Facilitators [12], coaches, resource providers, enablers, and motivators [32], knowledge facilitators [56], teaching facilitators [45], educational practitioners/facilitators [13], and facilitation processes in industrial setups [55]. These studies emphasize how facilitators play a crucial role in smoothing processes such as acquiring, buying, learning, and other essential activities in diverse fields. The healthcare facilitators can be individuals, such as general practitioners, counsellors, or organizations such as non-governmental organizations (NGOs), fundraising companies, working to improve the lives of people with chronic illness like cancer [15]. These organizations assist in identifying doctors, hospitals, and available policies or schemes during treatment, providing donors, channelling basic supplies, and more [4]. Research indicates that partnerships between NGOs (facilitators) and hospitals significantly improve cancer patient accessibility, especially in diverse, infrastructurally challenged, or psychosocially affected areas [2]. As mentioned above, Assam, has significantly high incidence of chronic disease in the North Eastern region. Therefore, this study will unpack NGOs working as health facilitators for chronic diseases, specifically cancer, located in the state of Assam, India and their role in supporting chronic disease.

Research indicates that the effectiveness of supplementary services can be examined using the Flower of Service model. According to the Flower of Service Model [35, 64], services consist of two components: the core service and the supplementary services. The core service delivers the fundamental benefit to consumers, while the supplementary services support the core service and add value to the overall customer experience. Sir Christopher Lovelock identified eight supplementary service clusters: information, order-taking, billing, payments, consultation, hospitality, safekeeping, and exception handling (special request handling). The first four are facilitating services, while the remaining four are enhancing services [34]. These supplementary services enhance core medical services by addressing the diverse needs of healthcare patients [21, 47]. In complex core services like healthcare for chronic diseases such as cancer, which involve long-term medical attention and ongoing treatments, it becomes imperative that supplementary services exist for the overall recovery of the patient and to smoothen the journey. For instance, when a patient pursues cancer treatment in a metropolitan city such as Chennai, Mumbai, Delhi, Kolkata, or Bangalore, they may either complete the full treatment course in that city or return to their home state, adhering to a treatment plan outlined by the city's doctors and continuing their care locally. For those seeking treatment outside Assam, accessing additional services like accommodation, doctor referrals, available schemes, and arranging blood donors can be particularly challenging in an unfamiliar environment. For marginalized sections of society, since diseases affect individuals regardless of their socioeconomic status, it is crucial to provide basic support such as flight allowances and take into account the patient's financial situation. Furthermore, when a patient chooses to continue treatment in their home districts, they still need help with accommodation, reducing additional expenses by supplying cooking gas, food, supplements, and more. Therefore, supplementary services are vital for the completion of cancer treatment but not all supplementary services are necessary for satisfaction, depending on the service type [47]. Numerous studies, including those on medical tourism [28, 48], the private hospital sector [27], tourism [40], banking industry [25], retailers and financial sector [14], application of artificial intelligence in Hospitality services [42] have applied this model, broadening our understanding of its use across various sectors.

The researcher attempts to investigate the use of the Flower of Service model by applying the supplementary service clusters proposed by Lovelock within the healthcare facilitator's context. This study uses Flower of Service model to examine how facilitators enhance core healthcare services by providing supplementary services for chronic disease patients' recovery. There is a need to understand how facilitator organizations in the healthcare sector work and offer services, highlighting a significant research gap about their contributions in Assam, India. Understanding the role of healthcare facilitators is essential for optimizing service utilization, as there is a lack of awareness about the existence of facilitator organizations among healthcare customers, underscoring a significant knowledge gap. It also seeks to understand what healthcare customers expect from these facilitator organizations. However, it is important to note that this study only uncovers the role of facilitator organizations in the healthcare sector, as the main medical service providers are not part of the study.

## Methodology

### Population, period of time, sampling technique

The facilitator organisations considered in the study are only non-governmental organizations (NGOs) operating in the healthcare sector and dedicated to addressing chronic diseases especially cancer. And Non-Governmental Organisations (NGOs) that are situated in Assam were considered for the study as facilitators.

According to the Central Statistical Institute of India in 2009, there were 3.3 million registered non-governmental organizations in India. However, pinpointing non-governmental organizations exclusively dedicated to chronic diseases is challenging, as no comprehensive list of these organizations could be located. Upon contacting the Registrar of Firms and Societies Assam, it was found that while there are NGOs listed based on timelines, no comprehensive list of NGOs specifically focusing on chronic diseases in Assam could be identified. Consequently, the exact size of the study population remains indeterminable hence the study used non-probability sampling. The study aims to comprehend and investigate the unique roles that FOs play in addressing these challenges in the northeastern Indian state of Assam. Additionally, the study examines the role that FOs play in the overall recovery of healthcare patients, making the study a blend of both descriptive and exploratory research design.

The snowballing procedure was used for the study as FOs were widely scattered and hard to reach throughout Assam. The process began with a participant who had over 15 years of operation, located in Guwahati, the capital city of Assam. After interviewing the first participant, they were asked to refer other contemporary FOs who met the study criteria. Each new participant, in turn, referred more FOs, creating a chain of referrals. This process continued until a sample size of nine FOs from Assam was achieved. During the snowball sampling process, we initially identified fourteen facilitator organizations (FOs) through referrals. However, only nine of these organizations were enrolled in the study. As the remaining five did not meet the inclusion criteria, the inclusion criteria were specifically focused on FOs addressing chronic diseases, such as cancer, in Assam. Organizations involved in other areas, such as community health, disabilities, sanitation, and so on, were excluded from the study. The facilitator organizations (FOs) that were traced were all NGOs operating solely on a non-profit model.

The "Flower of Service" model has been utilized for this research to understand how Facilitator Organizations (FOs) assist patients with chronic diseases by offering supplementary services. This research also aimed to explore patients' expectations from FOs in the context of managing chronic diseases such as cancer. To address these questions, the Lovelock model was used as a framework for constructing the questionnaire. The model proves to be instrumental in shaping the healthcare consumers' expectations of FOs and how the supplementary services provided by these organizations impact patients with chronic illnesses.

### Data collection and analysis

The data collection was done from April 2023 to August 2023 using questionnaires and both open-ended (open text) and closed-ended (5-point Likert, ranks, etc.) were part of it. Respondents included key informants from various facilitator organizations, who were interviewed for 30 to 90 min. Data was collected through Google Forms and a field diary was kept aside to note the interviews, as those who couldn’t be accessed physically data was collected electronically using Google Forms and telephonic interviews. The tools were prepared in both English and Assamese, the language spoken by the respondents, and later translated into English. In addition to the anticipated outcomes, a wealth of information emerged during these interactions, prompting the transformation of the questionnaire into an interview schedule. Descriptive statistics were used for quantitative data and qualitative data were classified into codes and iterative processes were used to deduct and induct the information until four major themes were derived. The field survey permit certificate, published by the educational institute as a part of the Ph.D. study was distributed to respondents either in person or via email, based on their accessibility. Informed and voluntary consent using a consent form (describing the nature, objective, purpose, potential risk and publication ethics of the research) was taken from all participants who were willing to be part of the research. Throughout the research, the identities of participants were kept confidential (pseudonyms were given).

### Participants

The interview was conducted with the key informants in the respective facilitator organisations (including Honorary secretory, Director, Vice president, etc.). Notably, the study excluded healthcare service providers focusing on core services, concentrating solely on those offering supplementary services (Table 1).

**Table 1:** Overview of the interviewees and data sources

Organisation	Interviewee/ position	Background/nationality	Other data source
FO1	Honorary General Secretary	Non-medical / academician/ Indian	Website/ social media page /online community page/direct/ observation
FO2	Director	Non-medical/ Retired Principal/NRI(Non resident Indian)^a^	Website/ newspaper/
FO3	Chief Executive Officer	Non – Medical/ Doctoral candidate/ Social Worker/Indian	Website/ friends / newspapers/ pamphlet/ television
FO4	-	Non-medical / Indian	Website/ newspaper/ television
FO5	Assistant Manager- Corporate affairs	Non-medical/ corporate personal / Indian	Website/ blogs/ newspapers/ friends and colleagues
F06	-	Non-medical / Indian	Website / newspaper / television
FO7	Vice president	Medical background/ Social Worker/ Indian	Referral through FO5/ Social media/ Websites/ television
FO8	Centre Head	Non-medical / Indian	Referral through F01/ online reading communities like ‘Granth Hubakh’/ television/ friends and family
FO9	Programme Manager	Medical background/ Indian	Referral through FO1/ Website

(Source: Field Survey)

^a^An NRI is someone who spends less than 182 days in India during a financial year and less than 60 days in the specific previous year, while also staying in India for fewer than 365 days in the preceding 4 financial years. NRIs can be Indian citizens or persons of Indian origin (PIOs).A Non-Resident Individual is not a resident of India for tax purposes. The determination of residential status is based on Sect. 6 of the Income Tax Act, 1961 [24]

## Findings

### Customers expectation from the facilitators

Approximately majority of healthcare customers, as reported by the FOs (FO1,2,3,4,5,8,9) turn to facilitator organizations for guidance before making their choice of a hospital. This underscores the fact that individuals dealing with chronic diseases in healthcare often rely on these facilitator organizations as a valuable resource in their treatment journey [31].

*A 14-year-old son of a family was diagnosed with cancer and they had to travel to Bangalore from Assam for advanced treatment. The family stayed there for almost a year, navigating an unfamiliar city with different food, culture, and language. Initially, they struggled with accommodations, transportation, language barriers and finding treatment guidance. By staying in contact with our organization, they received support through our extensive network, especially when they were unable to find blood donors. (FO9).*

Several organizations, including FO1, FO5, FO7, FO8, and FO9, primarily specialize in offering accommodation services for a very minimal or no price. As noted by Song [54], patients often face financial burdens, but involving facilitator organizations (FOs) instead of private options can reduce the cost. The data from the field shows that the primary goal of these organisations is to diminish reliance on private players in this process, leading to the emergence of facilitating organizations.

*During my mother's OPD session, I met a girl who was with her mother for a doctors visit. She suggested that we take lodging at FO9, as they offer free accommodation with basic amenities. My mother and I decided to leave the private lodge, which was charging us Rs 800, and move to FO9. This decision has saved us a lot of resources, and my mother was happy there. (P37).*

Customer expectations in healthcare, shaped by past and current experiences, significantly impact their perception of care quality [3, 69]. These expectations include desires for long-term relationships with providers, open communication, respect for opinions, and comprehensive care addressing both physical and mental health [23]. The World Health Organization highlights that integrated, patient-focused care can improve health outcomes, health literacy, patient and worker satisfaction, service efficiency, and reduce costs. Individuals with chronic conditions often have well-informed expectations based on their research, other’s experiences as well as their own.

*My parents initially didn't tell me about my condition (Acute Lymphoblastic Leukaemia) right after my diagnosis. I found out through online research and reviewing my reports, also I watched many YouTube vlogs of children with similar conditions. Through these videos, I learned that having to visit the hospital for follow-ups for the rest of my life will be the most challenging part of my journey. (P40- 12year old)*

### Relational longevity and continuity

Continuity in relationships enhances health management and outcomes for customers, especially those with complex health conditions [5]. Additional manpower for FOs to conduct rural outreach, systematic monitoring, chronic illness registries, and clinical outcome indicators can improve patient health outcomes accountability [17]. These measures are crucial for individuals with chronic illnesses, who require a variety of services to manage their conditions and symptoms even after the treatment period ends [54]. Consequently, families dealing with chronic diseases often engage with FOs over the long term.

*When we visited villages near Maridhal in Dhemaji to spread cancer awareness, it stemmed when a 52-year-old from the village approached us for accommodation, after being diagnosed with ovarian cancer. Upon asking what are the symptoms she experienced, stated that she had abnormal Uterine bleeding for five long months and finally decided to visit Assam Medical College, Dibrugarh. Our team decided to educate her and the neighbouring village about cancer and emphasize the importance of immediate doctor visits when they feel something wrong in their body and needs attention. (FO 9)*

Understanding how these patients perceive and feel about their interactions with FOs can enhance the quality of their recovery. Healthcare customers receiving quality care demonstrate better treatment adherence, improved self-management skills, greater motivation, and a positive, ongoing relationship with their FOs, all of which are essential for good patient outcomes.

*Sadness and worry are the biggest concerns of the family members towards the patient during their treatment (FO7).*

Hence, many key informants in the study emphasized the importance of long-term relationships with healthcare consumers for effective chronic disease management.

### Addressing disparities through holistic development

**Fig. 1:**
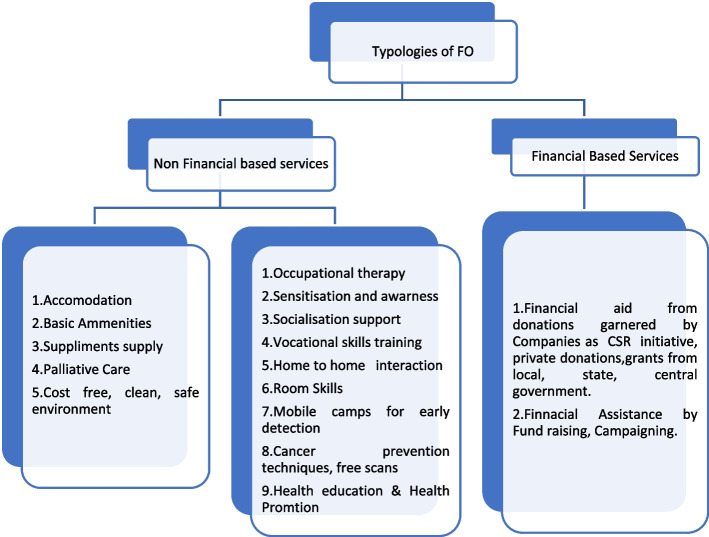
Typologies of FOs based on services they provide. (Source: Field Survey)

Cancer care is transitioning from a disease-focused to a patient-centred approach, addressing physical, psychological, social, spiritual, and financial concerns [11]. A comprehensive cancer help agency should aim to provide holistic patient care, aid, assistance, awareness, early detection, rehabilitation, and advocacy. This shift requires understanding the factors that exacerbate the cancer burden and the role of support organizations (FOs) [67, 68]. According to a study individualized treatment plans are crucial for offering the best chances of cure [43]. FOs play a crucial role by providing vocational skills, occupational therapy, and addressing long-term disabilities caused by treatment side effects, which often prevent patients from returning to work. Many incurable cancers, especially haematological ones, impact patients and caregivers physically, emotionally, and socially (Fig. 1).

*Patients while going through the high-dose and conventional chemotherapy regimens that render employment impractical for months, remain completely dependent on family, and external support and at one point necessitate advice on socio-economic issues from us ( FO9).*

**Fig. 2:**
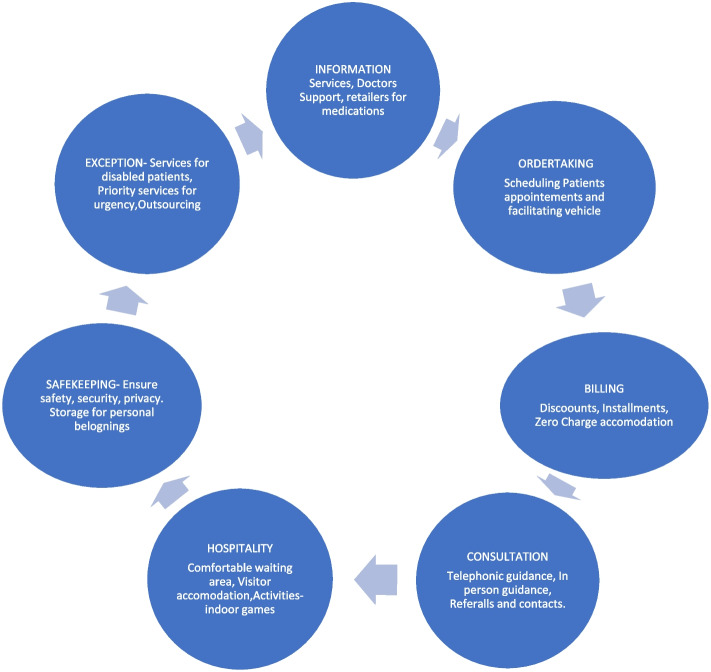
Application of FO’s services in Flower of Service Model (Source: Field Survey)

FOs assist with information on state benefits and social services, such as Disability Living Allowance and government schemes. Patients with cancer often face psychological disorders such as anxiety, depression, and PTSD, which can also delay their return to work [41]. Supportive care offered by FOs helps patients manage self-care, work productivity loss, distress, and unmet needs, in Assam, some FOs also provide palliative care. Although patients cannot reside in the facilitator's home, a team of doctors makes periodic visits to the patient's home for timely inspection making the end of life care easy. FO1, 2, 5, 8, 9 contribute to the holistic development of cancer patients by offering a range of supportive services. A set of typologies based on the portfolio of services provided has been developed, distinguishing between financial and non-financial services in Fig. 2. The financial services are further categorized into those offered during treatment and those provided after the completion of treatment for cancer patients.

### Effective communication

Effective communication is essential in the patient-physician relationship, crucial for patient-centred care and improved patient outcomes. According to Kourakos et al. [33], patient-centred communication enhances trust, satisfaction, chronic disease management, disease-specific outcomes, and quality of life during care and treatment. As one of them asserted.

*I feel that patients' feeling of being heard addresses a lot of emotional needs during chronic diseases and also gives them the assurance that alleviates the patient and their family from the sense of helplessness ( FO7).*

FOs empower patients by using therapeutic communication techniques like active listening, open-ended questions, and reflecting on patients' concerns, by infusing indoor games like Ludo, Chess during their stay in the facilitating homes for treatment. Building trust and involving patients in decision-making, along with providing information and support for self-management, are key strategies in recovery and care. Hence, initiating relationships with honesty, openness, compassion, and respect is vital for establishing trust among them. For patients with chronic diseases, understanding and addressing their specific challenges is important, as they may exhibit defensive behaviours that hinder communication.

*Patients and their families receive free treatment at government hospitals and accommodation with basic amenities in the Facilitator's Home. However, when it’s time for them to return to their villages, they often find themselves without money for transportation. Despite this, they are so principled that they refuse to accept financial assistance. To address this, our organization helps by finding daily wages or suitable work in nearby areas so that the family can support themselves (FO9).*

It highlights how families with chronic illnesses are often determined to help themselves and remain independent, even when facing challenges.

While being in the facilitating organisation proper hygiene, including oral care, bathing, and grooming, is crucial for maintaining patient health and preventing complications [22]. According to FO8 and FO9, maintaining hygiene is a key factor in the recovery process. These FOs assist patients with activities like bathing, bed bath, dental care, elimination etc. As neglecting these aspects can lead to adverse effects such as aspiration pneumonia, opportunistic infections [37, 38], gram-negative infections [26], and Clostridium difficile infections [46].

*The underprivileged patients at our centre often lack basic hygiene, discarding leftover food, including lemons and bones, into the lavatory seats. This practice causes food to be trapped in the outlets, disturbing other patients. During difficult times, it becomes challenging for everyone to address these minor issues. Our team repeatedly intervenes to educate them about the importance of maintaining hygiene and explain how poor hygiene can harm their health and that of their fellow patients (FO9).*

FO9 states that some patients may prefer advice from facilitators over doctors, particularly on hygiene and cleanliness. Thus, healthcare providers should adopt a patient-centred approach, tailoring their communication to each patient's unique needs and preferences. This includes using simple, patient-specific language, maintaining a clear and calm tone, and respecting patients' decisions, even when they differ from medical recommendations.

### Therapeutic support

#### Mental health

Providing social support after a patient is discharged from a healthcare facility is crucial for their recovery and overall well-being [66]. Patients from lower socio-economic groups tend to have worse health outcomes compared to their wealthier counterparts, partly due to a lack of social interactional support [59]. The absence of support often leads to feelings of being ignored and is associated with loneliness. Patients frequently display social avoidance behaviours such as withdrawing from the outside world or refusing to share their concerns with others [44]. Consistent with the findings, recent studies indicate that some patients perceive cancer as originating from supernatural forces, curses, or punishment from lesser gods or ancestors for past wrongdoings [1]. Therefore, facilitators (FO1, FO3, FO8) advise patients not to wander around their villages, as villagers still believe that cancer is a consequence of sins. However, the facilitators are uncertain whether patients follow this guidance once they return to their villages from the facilitators' homes.

*Often patients with chronic diseases are seen with a sympathetic gaze or them being helpless and dependent or being a burden on their families. So, we advise patients to avoid wandering around their village or interacting with the public to prevent stigmatization and negative comments from villagers. As these cautions are crucial because some villagers still view chronic diseases as taboo, attributing them to perceived past sins or karma associated with the patients and their families (FO8).*

FO1,3,7,8,9 focuses on giving caregiver education to empower family members or caregivers with the knowledge required to comprehend the patient's condition, manage their medications, and provide emotional support. Healthcare facilitators ensure attendants understand their family members medications, including proper dosages and timing, and recommend tools like pill organizers or mobile apps to aid adherence.

*Before releasing patients from our facilitator's home, we train their attendants on providing timely medication, using mobile applications for pill management, and teaching room skills, emphasising leading healthy lifestyles and the importance of follow-ups.( FO8)*

### Infusion of occupational therapy

Cancer survivors often express facing challenges in all aspects of life due to their treatments [7] and can worsen the physical, cognitive, and emotional aspect, impairing function and limiting activities [8, 50] reducing survivors' self-efficacy and independence.

*We provide livelihood support by teaching social, vocal, and room skills. We reinstall skills in patients who have suffered for a long time, providing cattle or weaving machines (Taat haal) based on their interests and expertise. We offer vocational training to patients over 18 years old and implement various income generation models, such as piggery units, with incentives distributed model etc. at an later phase as this approach empowers our patients (FO8).*

For cancer survivors, occupational therapy can be crucial because it helps patients deal with and get past the psychological, cognitive, and physical difficulties. Thus, from the investigation it was found that FO9 emphasises health care customers to participate in everyday occupations of life assisting in the restoration of everyday functioning and independence allowing survivors to resume their in tasks such as self-care activities including showering, dressing, preparing food; imparting education, learning to work, emphasising and caring for others.

### Provision of rehabilitation

WHO 2024 defined rehabilitation as a set of interventions designed to optimize functioning and reduce disability in individuals with health conditions in interaction with their environment [62]. It is a type of care designed to help individuals recover, sustain, or enhance their daily living skills [39]. By restoring their abilities, rehabilitation allows people to return to or engage more fully in family, work, educational, and community activities, which can have significant economic and social benefits [62].

For instance FO1’s commitment to rehabilitation extends not only to patients but also to their families, as demonstrated by an incident shared by the Honorary Secretary of FO1:

*There was a family where the father worked as an auto driver. However, due to the financial strain caused by their son's chronic illness, they had to sell their auto, which was their primary source of livelihood. The son eventually recovered from the illness, and the family returned home from Mumbai. However, they found themselves in a desperate situation as they had lost their means of earning a living. Upon learning of the family's predicament, our team took action, shared the family's story within the network, and this led to a generous donor coming forward. The donor expressed a desire to help and offered to donate an auto to the family* (FO1).

Majority of the FOs demonstrates this approach to rehabilitation and community support, recognizing that the well-being of the entire family is essential for the overall recovery. Rehabilitation restores functioning in patients, helping them return to their family, work, education, and community roles, potentially with minimal economic and social implications.

As the patient's journey is complex and the need to redevelop social skills, rehabilitation is of paramount importance. It not only enhances cognitive and emotional functioning, but also improves work capacity, reduces health-related issues, and contributes to the overall recovery of both the individual and their family emphasized [10, 36].

### Supplementary services offered by facilitator organisations

The study reveals that Facilitator Organizations (FOs) offers a variety of supplementary services to healthcare customers by using their communication skills to guide patients in choosing suitable healthcare facilities. To fully benefit from these services, customers requires relevant information, such as directions to the facility, who referred them, opening hours, services offered, pricing, and rules [64]. Healthcare customers expect FOs to be knowledgeable and information-rich [42]. The primary role of FOs is to provide information and respondents also indicated that patients and their attendants frequently contact FOs for basic information, such as the availability of doctors in Assam for specific diagnoses. FOs reported that most inquiries come from parents of children with various types of blood cancer, asking about the number of available doctors and the cost of treatment. Notably, healthcare customers maintain long-term relationships with FOs, later seeking assistance for blood donors, among other needs.

In addition, order-taking involves onsite or online entries, reservations, and application completions for services [64]. Billing entails informing customers of the due amount, billing methods, and improving the billing process [64]. For example, that data analysis shows travellers using online travel agencies can view the total cost on a website or app, with billing statements emailed as payment reminders. For underprivileged patients, FOs often provide free or heavily discounted accommodation and coordinate with suppliers or donors for supplements needed due to treatments like chemotherapy. Payments in the Flower of Service model involve payment methods, parties, and security issues [64]. As FOs operate on a non-profit model, making the ‘payment’ function less significant, nominal fees are usually paid in cash, with minimal use of digital payments.

Additionally, Consultations involve dialogues between customers and FOs to understand customer needs, resulting in direct suggestions or solutions through counselling [64]. Consultations are conducted either over the phone or in person.

Furthermore, safekeeping ensures the care of customers and their possessions [64]. FOs provide proper bedding and lodging with good security measures in place, offering both rooms and dormitory services monitored by staff. Hospitality involves caring for new and returning customers, including greeting, providing food, waiting facilities, and transport [64]. Most FOs have waiting rooms equipped with chairs, tables, ceiling fans, round tables, and a proper ambiance. FOs provide proper hospitality at facilitator homes where patients are accommodated.

In conclusion, exceptions address special customer requests and unexpected circumstances, such as compensations after service failures [64]. For example, a breast cancer patient once experienced severe bleeding at a facilitator's home (FO1), requiring external nursing services. This incident underscores the need for FOs to outsource specialized medical services to handle unfamiliar situations effectively. Thus, information, safekeeping, consultation, hospitality, exceptions are crucial supplementary services, with FOs playing a significant role in patient advocacy and addressing special requests whereas payment and billing is less effective in case of FOs that works for chronic diseases patients. Hence, a model was developed to denote the set of supplementary services that are utilised in the FO’s context using Lovelock's original Flower of Service model [35] and the order of the petals follows this. However, this figure omits the 'payment' petal, as noted by FO1, 2, 4, 8, and 9, which indicate that they provide services free of charge and do not have any provisions related to payment (Fig. 2).

## Discussion

This study identified the services provided by Facilitator Organizations (FOs) and their significant impact on the overall recovery of healthcare customers, categorizing them into four major themes. Firstly, relational longevity and trust-building are crucial for patients in recovery, as they heavily depend on these FOs. According to a study by Song et al. [54], individuals with chronic conditions do not base their experiences solely on individual clinical interactions. Instead, their experiences are shaped by an ongoing relationship with their service provider, where expectations are communicated and appropriately responded to over time. FOs such as FO1, FO7, FO8, and FO9 focus on maintaining relational continuity with healthcare customers. However, FOs like FO2, FO3, FO4, FO5, and FO6 specialize in offering financial help. FO3 particularly works on organ donation, linking donors and patients who typically engage in one-time transactions or assistance.

In addition, FOs address the individual needs of patients holistically and in a patient-centred manner as is crucial for those living with and beyond cancer. Holistic care, which addresses the effects of cancer treatment, risk factors associated with recurrence, and other physical and spiritual needs, remains a significant unmet necessity and is becoming increasingly important [52]. This study developed a typology of Facilitator Organizations (FOs) based on the range of services they offer, providing a holistic approach to patient recovery, these services are divided into financial and non-financial categories. Non-financial services are further categorized into those provided during treatment, such as accommodation, basic amenities, supplement supply, palliative care, and a safe environment, and those needed during or after treatment requiring long-term attention and guidance, such as occupational therapy, sensitization and awareness, social support, vocational skills training, mobile camps for early detection, cancer prevention techniques, free scans, and health education and promotion. For financial services, FOs provide two types: financial aid from donations through CSR initiatives, private donations, and government grants, and financial assistance through fundraising and campaigning.

Effective communication is crucial in healthcare customer interactions, requiring honesty, openness, compassion, and respect. Understanding and addressing the specific challenges faced by patients with chronic diseases, who may exhibit defensive behaviours that hinder communication, is particularly important. The quality of patient-physician communication significantly influences patient-centred outcomes [16]. FOs like FO1, FO2, FO3, and FO4 utilize tools to measure and improve effective listening, employing techniques such as listening, smiling, making eye contact, eyebrow movements, and telephone communication with patients.

In addition, FO1, FO2, FO7, FO8, and FO9 provide both physical and mental health support through therapeutic treatments like palliative care, occupational therapy, complementary therapy, and back-to-school measures. These organizations also focus on patient education, offering valuable information on cancer management and legal assistance. Furthermore, FOs advocate for patient rights and strive to improve healthcare services, contributing to a more supportive and effective cancer care environment.

Earlier the delivery of the core and supplementary services was done by different service departments and employees associating large manpower, in health care services [27]. According to Raydback 2022, hospitals handle the provision of core services, while Medical Tourism Facilitators (MTM) handle supplementary services like information dissemination, support industry, consultation facilitators, hospitality providers, reservation agents, network developers, etc. According to Raydback et al. [48], the fact that these services are sought after suggests that these intermediaries fill a need that core suppliers are unable to. All of the Flower of Service model's components were shown to be helpful in the setting of healthcare facilitators in this study, with the exception of the ‘payment’ component, which is less significant because these FOs run on a nonprofit basis. This proves that the existence of these FOs by providing supplementary services enhances the core hospital services.

This finding of this research provides valuable first-hand information and insights, closely reflecting the real-world experiences of individuals seeking healthcare services for chronic conditions, which require ongoing medical attention. FOs operate within a complex framework that necessitates both medical and non-medical expertise, working alongside medical professionals to significantly contribute to the overall well-being of healthcare consumers and their families. The limited number of FOs despite the high annual cancer diagnosis rate in the NE region, India, restricts geographic diversity, presenting an opportunity for future research to explore geographic variations and regional vulnerability and gaps as well. Additionally, there is a scarcity of FOs dedicated not only to chronic diseases but also to addressing various health conditions. As the study focuses on FOs managing chronic diseases, the findings may not be generalizable to those dealing with acute conditions. Finally, access to clinical trials and advanced treatments or provisions of hospice care is not offered by the present FOs in Assam, further indicating areas for future investigation.

## Conclusion

The study explored the role of Facilitator Organizations (FOs) in healthcare, with a focus on chronic diseases, and found that these organizations are crucial in supporting cancer patients in Assam. FOs in Assam offer a wide range of services tailored to the unique needs of patients with chronic conditions, highlighting their essential role in the recovery process. However, a comprehensive list of NGOs specifically focused on chronic diseases was not available in the state, this suggests that while the number of FOs is limited, these organizations are crucial for amplifying patient voices in policymaking, boosting grassroots mobilization, improving health systems, and supporting regional cancer care goals. Integrating both core and supplementary services within health systems is vital for meaningful and sustainable patient engagement not only in Assam but also in the country. The study finally highlights a growing need for more FOs in Assam due to the state's high rate of cancer diagnoses.
